# Semi-Supervised Traffic Sign Detection with Dual Confidence Fusion Module and Structured Block-Regularized Neck

**DOI:** 10.3390/s26051601

**Published:** 2026-03-04

**Authors:** Chenhui Xia, Yeqin Shao, Meiqin Che, Guoqing Yang

**Affiliations:** 1 School of Transportation and Civil Engineering, Nantong University, Nantong 226019, China; 2 Suzhou Research Institute of Industrial Technology, Zhejiang University, Hangzhou 310058, China

**Keywords:** traffic sign detection, semi-supervised learning, Dual Confidence Fusion Module, Structured Block-Regularized Neck network, pseudo-label optimization, Spatial-Context-Aware Upsampling

## Abstract

Reliable traffic sign detection is essential for the safety of autonomous driving systems. However, manually annotating large-scale datasets for this task is resource-intensive, making semi-supervised learning (SSL) a vital alternative. Despite their potential, current SSL methods often struggle with unreliable pseudo-label filtering and limited detection accuracy. To address these limitations, we propose a novel framework integrating a Dual Confidence Fusion (DC-Fusion) module and a Structured Block-Regularized Neck (SBR-Neck). The former improves pseudo-label reliability by combining classification and localization confidence scores, while the latter optimizes feature representation through multi-scale fusion and block-wise regularization. To preserve high-frequency spatial details, SBR-Neck incorporates Spatial-Context-Aware Upsampling (SCA-Upsampling), which utilizes multi-granularity feature decomposition. Experimental results on a proprietary traffic sign dataset demonstrate that our method achieves ${\mathrm{mAP}}_{50}$ scores of 10.4%, 17.8%, 23.7%, and 32.1% using 1%, 2%, 5%, and 10% labeled data, respectively. These results surpass the “Efficient Teacher” baseline by margins ranging from 3.07% to 11%, confirming the framework’s ability to provide robust detection in complex traffic scenarios.

## 1. Introduction

Traffic signs provide key visual information to drivers, while misinterpretation or missed detection of traffic signs can lead to serious traffic accidents. Recently, the rapid development of Intelligent Transportation Systems (ITSs) has significantly enhanced urban traffic management and efficiency [1]. In particular, advanced cooperative control architectures for connected and automated vehicles, such as the MAS-based hierarchical cooperation framework proposed in [2], demonstrate the increasing intelligence and system-level integration in modern transportation systems. In this context, with the advancement of autonomous driving and advanced driver-assistance systems (ADASs), accurate detection of traffic signs has become increasingly critical to guarantee driving safety [3].

Deep learning-based object detection methods have achieved great breakthroughs in recent years, mainly thanks to the availability of large-scale labeled datasets [4,5,6,7,8,9]. These fully supervised approaches have shown excellent performance in various domains. However, their dependence on extensive human annotation leads to significant limitations, as the process of labeling is time-consuming and expensive in terms of finance and human effort, especially for traffic sign detection with high-accuracy requirements.

To reduce dependence on labeled data, researchers have turned to semi-supervised learning methods [10,11,12,13,14], which depend on a small set of labeled data and a large pool of unlabeled data. By exploiting the information of unlabeled data, semi-supervised learning can achieve accurate and robust detection results with much less dependence on costly annotations, especially when labeled data is difficult to obtain.

Recently, semi-supervised deep learning methods have shown promising experimental results in traffic sign detection, making better use of large amounts of unlabeled data [15,16,17,18,19,20,21,22,23,24,25]. Among these methods, pseudo-labeling has attracted significant attention, where a teacher model generates pseudo-labels for unlabeled data, which are then used to train a student model.

While traditional methods rely on classification confidence for pseudo-label filtering, recent confidence-aware SSOD approaches, such as Soft Teacher [26] and Unbiased Teacher [27], have attempted to assess localization reliability using box jittering or uncertainty estimation. However, these methods generally estimate localization quality through statistical variance, which serves as an indirect and computationally intensive proxy rather than a reflection of instance-level geometric accuracy. They lack a unified and explicit metric to jointly evaluate the reliability of semantic classification and geometric localization, leading to localization errors that propagate into the pseudo-label assignment process. Furthermore, the feature fusion strategies in current frameworks often fail to fully exploit multi-scale image features, limiting generalization ability, especially for long-distance traffic signs. By comparison, our DC-Fusion establishes a dual-branch structure that outputs classification confidence and IoU prediction. By utilizing the product of these two metrics as the final confidence score, our method introduces a missing, unified, and explicit metric by directly binding semantic probability with geometric precision. This avoids reliance on indirect statistical proxies to directly evaluate instance-level accuracy, thereby addressing the problem that high classification scores are often accompanied by inaccurate localization.

To address these limitations, we propose a novel semi-supervised traffic sign detection framework with Dual Confidence Fusion and Structured Block-Regularized Neck. Based on the lightweight and efficient end-to-end architecture of YOLOv11, our approach leverages its fully convolutional one-stage design to directly predict object classes and bounding boxes without region proposals, enabling real-time inference. The proposed framework further incorporates a dual-branch confidence fusion strategy and structured feature enhancement modules to improve pseudo-label quality and enhance the detection robustness of long-distance traffic signs under limited supervision. Our main contributions can be summarized as follows:

1. To improve the reliability of pseudo-labels, we design a Dual Confidence Fusion Module (DC-Fusion) that jointly integrates classification and localization confidences of each object into a unified scoring mechanism for more accurate filtering of high-quality pseudo-labels. We introduce a refined positive–negative–ambiguous sample assignment strategy, mitigating the negative effects of noisy pseudo-boxes and enhancing the robustness of the student model during training.
2. To enhance detection performance for long-distance traffic signs, we propose a Structured Block-Regularized Neck network (SBR-Neck) to replace the conventional neck network. SBR-Neck incorporates multi-level feature fusion and block-wise spatial perturbation, which regularizes local activations and strengthens the generalization of learned representations.
3. To preserve spatial details of traffic signs during feature upsampling, we introduce a Spatial-Context-Aware Upsampling (SCA-Upsampling) operation. This operation explicitly models spatial context by multi-granularity feature decomposition and depthwise separable convolution, thus improving localization accuracy for long-distance or low-contrast traffic signs.

Experimental results on our self-built traffic sign detection dataset demonstrate that the proposed method consistently outperforms state-of-the-art semi-supervised detection models. In particular, under the 10% labeled data setting, our method achieves a mAP_50_ improvement of 7.5% over the baseline model (Efficient Teacher), proving the effectiveness of our proposed method.

## 2. Related Work

### 2.1. Traffic Sign Detection

Traditional methods for traffic sign detection typically follow a two-stage pipeline [28]. In the first stage, candidate regions that potentially contain traffic signs are generated based on hand-crafted features such as color, shape, or edge information. This stage aims to significantly reduce the search space by filtering out irrelevant background regions. In the second stage, these candidate regions are further analyzed using machine learning classifiers such as Support Vector Machines (SVMs) or AdaBoost, which are often trained on discriminative features like the Histogram of Oriented Gradients (HOG) [29,30]. This two-step process enables traditional methods to achieve basic detection capabilities, but their heavy reliance on manually designed features often limits robustness under complex road environments.

With the advent of deep learning, Convolutional Neural Networks (CNNs) have outperformed traditional methods by learning features directly from raw data [31,32]. Wang et al. [33] proposed an improved feature pyramid network, which incorporates an adaptive attention module and a feature enhancement module. Omid et al. [34] introduced a pyramid transformer that combines local attention with a hierarchical structure to capture both local and global features. Their method uses multi-head self-attention with residual connections to enhance global feature extraction. Wang et al. [35] designed a context-aware and attention-driven weighted fusion network. It leverages multi-head self-attention and convolutional layers to extract contextual features and applies relative positional encoding for improved spatial representation. A spatial attention mechanism is used to enhance important regions during feature fusion. Zhang et al. [36] proposed a cascaded R-CNN framework using a multi-scale attention mechanism within a feature pyramid. This design focuses on highlighting traffic sign features across different resolutions and refines detection results through a multi-stage cascade process. These methods reflect recent studies in combining multi-scale representation, attention mechanisms, and context modeling to improve the accuracy and robustness of traffic sign detection models.

### 2.2. Semi-Supervised Object Detection

In semi-supervised object detection (SSOD), the ideas of self-training and consistency-based methods derive from semi-supervised image classification. A representative example is the Noisy Student framework, which inspired the design of STAC [37]. STAC uses a multi-stage process where a fixed teacher model first generates pseudo-labels for unlabeled data, and a student model is then trained using these labels. To address the limitations of this disjointed training process and improve pseudo-label quality, other SSOD frameworks [19,26,27,38,39] have been proposed. These methods typically adopt an Exponential Moving Average (EMA) to dynamically update the teacher model based on the student model’s parameters, which enables high-quality pseudo-labels to be generated during training. Within this framework, research has expanded from single-stage to more complex two-stage detectors. For instance, Unbiased Teacher [27] applies focal loss calibration to reduce bias in pseudo-labels, while Instant-Teaching [19] encourages the student and teacher to correct each other’s errors, reducing confirmation bias. Humble Teacher [38] introduces soft pseudo-labels for smoother knowledge transfer, and Soft Teacher [26] improves pseudo-label reliability by score-weighted classification loss and box jitter techniques. More recently, SSOD has extended to transformer-based object detectors. Semi-DETR [39] adapts DETR to the semi-supervised setting by introducing stage-wise hybrid matching to combine one-to-many and one-to-one assignment strategies, cross-view query consistency to maintain semantic consistency across data augmentations, and cost-based pseudo-label mining to filter out unreliable pseudo-boxes. Based on these methods, our work focuses on addressing selection and assignment ambiguities within the single-stage detector-based semi-supervised learning framework.

## 3. Materials and Methods

### 3.1. Overview of Our Method

**Figure 1:**
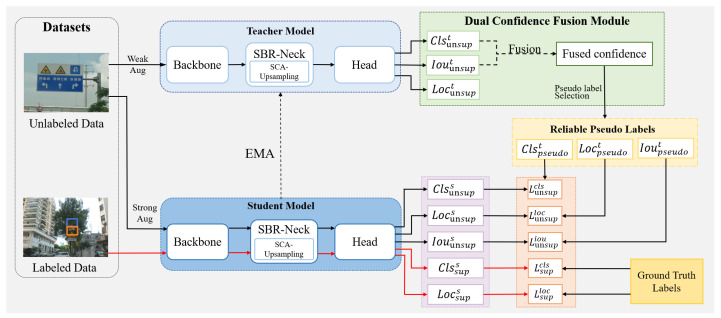
The overall architecture of the proposed semi-supervised traffic sign detection framework, which integrates the Dual Confidence Fusion Module (DC-Fusion) for robust pseudo-label generation and the Structured Block-Regularized Neck (SBR-Neck) to improve feature fusion and spatial localization. The framework builds on YOLOv11 and enables effective knowledge transfer from limited labeled data to a large pool of unlabeled data.

The framework of our semi-supervised method for traffic sign detection, based on the Dual Confidence Fusion Module and the Structured Block-Regularized Neck, is illustrated in Figure 1. First, the original dataset is divided into labeled data $X_{L}={\left\{x_{i}^{l}\right\}}_{i=1}^{N_{l}}$ and unlabeled data $X_{U}={\left\{x_{i}^{u}\right\}}_{i=1}^{N_{u}}$, where $N_{l}$ and $N_{u}$ denote the numbers of labeled and unlabeled samples, respectively, and $N_{u}\gg N_{l}$ typically. The labels for the labeled samples are denoted as $Y_{L}={\left\{y_{i}^{l}\right\}}_{i=1}^{N_{l}}$, each containing the center coordinates, width, height, and class information of the target bounding box.

To further enhance the effectiveness of our semi-supervised framework, we replace the original YOLOv5 detector with the advanced YOLOv11 architecture in both the teacher and student models, due to the specific challenges of semi-supervised traffic sign detection addressed in this work. Semi-supervised learning is highly sensitive to pseudo-label noise; hence, a backbone network that can obtain more accurate classification and localization predictions is essential. The attention-enhanced C3k2 module in the YOLOv11 backbone enhances feature discrimination and spatial modeling, improving the reliability of fused confidence estimation in our DC-Fusion module and reducing error propagation during teacher–student model training. Moreover, its strengthened multi-scale feature representation further helps in detecting long-distance traffic signs, complementing the proposed SBR-Neck and SCA-Upsampling.

To address ambiguity in pseudo-label selection and assignment, the Dual Confidence Fusion Module adopts a dual-branch structure that combines the classification response ${\mathrm{Cls}}_{\mathrm{unsup}}^{t}$ and the localization quality score ${\mathrm{Iou}}_{\mathrm{unsup}}^{t}$, obtaining a fused confidence vector defined as ${\mathrm{Conf}}^{t}={\mathrm{Cls}}_{\mathrm{unsup}}^{t}\cdot {\mathrm{Iou}}_{\mathrm{unsup}}^{t}$. A dynamic threshold is then applied to divide the sample set into positive, ambiguous, and negative samples. The positive samples are directly used for classification and localization tasks. The ambiguous samples are employed as follows: for classification, all ambiguous samples are trained via consistency learning to mimic teacher responses; for localization, only spatially aligned ambiguous samples are selected, and their target bounding boxes are generated by weighted averaging of high-confidence samples. The negative samples are ignored.

To enhance multi-scale feature fusion efficiency and prevent overfitting in YOLOv11, we design the Structured Block-Regularized Neck network (SBR-Neck) to replace the standard neck network. SBR-Neck builds upon cross-level feature interactions inspired by the Generalized Feature Pyramid Network [40] and introduces block-wise spatial perturbations to regularize feature activations and enhance global representation robustness.

In addition, to enhance the ability of upsampling to model spatial contextual information in the feature map and to address the problem of degrading object detail, we utilize the Spatial-Context-Aware Upsampling operation, which explicitly separates feature representations at different semantic levels via multi-granularity feature decomposition to perform high-resolution reconstruction.

During training, the teacher model is first pre-trained using labeled data, and its weights are copied to initialize the student model. The teacher model processes weakly augmented images to generate pseudo-labels, while the student model learns from strongly augmented inputs to improve robustness.

### 3.2. Dual Confidence Fusion Module

Most semi-supervised methods employ a classification score threshold to filter reliable pseudo-labels, but these methods only consider classification confidence while ignoring localization quality, which leads to unreliable pseudo-labels. To address this, we design the Dual Confidence Fusion Module, which integrates classification and localization confidences, and further introduces a neighborhood-based consistency check on ambiguous samples to improve pseudo-label selection and optimize positive–negative sample assignment.

**Figure 2:**
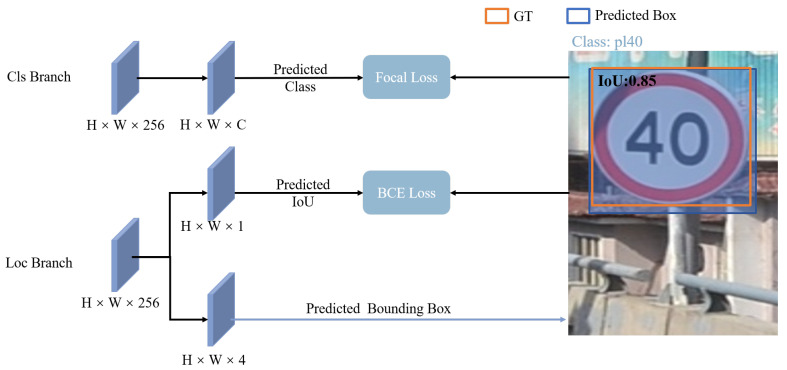
Detailed illustration of the Dual Confidence Fusion Module, featuring a dual-branch architecture where the classification branch predicts the class confidence vector and the localization branch estimates the bounding box and IoU score. The class confidence and IoU scores are fused to compute a joint confidence score for pseudo-label filtering, which improves sample selection accuracy and pseudo-label reliability in semi-supervised learning.

The primary objective of this module is to compute a fused confidence score to guide pseudo-label selection. As illustrated in Figure 2, DC-Fusion adopts a dual-branch structure: (1) a primary classification branch for object class prediction, and (2) an auxiliary branch for localization quality estimation. The black arrows represent the computation flow of joint confidence, which merges the outputs from the two branches. The resulting joint confidence score is used as supervision for both classification and IoU prediction with focal loss and binary cross-entropy loss, respectively. Meanwhile, the blue arrows indicate the prediction of the bounding box and its corresponding IoU score, which are also utilized to generate soft labels for training.

The fused confidence vector ${\mathrm{Conf}}^{t}$ is obtained by combining class confidence ${\mathrm{Cls}}_{\mathrm{unsup}}^{t}$ and localization quality ${\mathrm{Iou}}_{\mathrm{unsup}}^{t}$ predicted by the teacher model:

(1) $${\mathrm{Conf}}^{t}={\mathrm{Cls}}_{\mathrm{unsup}}^{t}\cdot {\mathrm{Iou}}_{\mathrm{unsup}}^{t},$$

where ${\mathrm{Cls}}_{\mathrm{unsup}}^{t}$ denotes the class probability distribution produced by the classification branch of the teacher model and normalized via a Softmax function. ${\mathrm{Iou}}_{\mathrm{unsup}}^{t}$ is the self-assessment score of the teacher model for its own positioning accuracy, which serves as pseudo-positioning confidence in unlabeled data. The fused confidence ${\mathrm{Conf}}^{t}$ thus encodes both semantic confidence and localization reliability for each class, and is used to guide the selection of pseudo-labels in the unsupervised branch.

We subsequently assign labels based on the fused confidence score produced by the teacher model, rather than depending on unreliable pseudo-boxes, as the fused confidence score more accurately reflects sample quality. The samples with very high or low confidence can be readily classified as positive or negative, respectively, while those samples with intermediate confidence remain ambiguous. Therefore, these ambiguous samples are selected for classification and localization tasks, respectively, as the sensitivities of different tasks to label uncertainty are different.

Specifically, we categorize each unlabeled sample into negative, ambiguous, or positive pseudo-labeled categories based on a fixed negative threshold $\tau_{\mathrm{neg}}$ and a dynamically computed positive threshold $\tau_{\mathrm{pos}}$:

(2) $$x_{i}=\left\{\begin{array}{cc} \mathrm{Negative}\;\mathrm{sample}, & \mathrm{if}\;\mathrm{Max}\left({\mathrm{Conf}}_{i}^{t}\right)<\tau_{\mathrm{neg}}\\ \mathrm{Ambiguous}\;\mathrm{sample}, & \mathrm{if}\;\tau_{\mathrm{neg}}\leq \mathrm{Max}\left({\mathrm{Conf}}_{i}^{t}\right)\leq \tau_{\mathrm{pos}}.\\ \mathrm{Positive}\;\mathrm{sample}, & \mathrm{if}\;\mathrm{Max}\left({\mathrm{Conf}}_{i}^{t}\right)>\tau_{\mathrm{pos}} \end{array}\right.$$

where ${\mathrm{Conf}}_{i}^{t}$ denotes the fused confidence vector produced by the teacher model for the *i*-th unlabeled sample. The operator Max(·) returns the maximum fused confidence score among the C dimensions; this score serves as a unified indicator for evaluating the reliability of the pseudo-label.

In this setting, the negative samples mainly refer to false positives with very low fused confidence and are therefore treated as background objects during the training procedure. The negative threshold $\tau_{\mathrm{neg}}$ is fixed to 0.1, and the positive threshold $\tau_{\mathrm{pos}}$ is dynamically computed in each batch based on the statistical distribution of the maximum fused confidence score over ambiguous samples and positive samples:

(3) $$\tau_{\mathrm{pos}}=\mu_{\mathrm{Max}}+\sigma_{\mathrm{Max}},$$

where $\mu_{\mathrm{Max}}$ and $\sigma_{\mathrm{Max}}$ denote the mean and standard deviation of the maximum fused confidence scores from the ambiguous and positive samples in the current batch, respectively.

After dividing the unlabeled samples into positive, ambiguous, and negative sets based on the fused confidence, the teacher model provides pseudo-labels that serve as targets for subsequent tasks. The pseudo-labels are defined as

(4) $${\mathrm{Cls}}_{\mathrm{pseudo}}^{t}={\mathrm{Cls}}_{\mathrm{pos}}^{t}\cup {\mathrm{Cls}}_{\mathrm{amb}}^{t},$$

(5) $${\mathrm{Loc}}_{\mathrm{pseudo}}^{t}={\mathrm{Loc}}_{\mathrm{pos}}^{t}\cup {\mathrm{Loc}}_{\mathrm{refined}}^{t},$$

(6) $${\mathrm{IoU}}_{\mathrm{pseudo}}^{t}={\mathrm{IoU}}_{\mathrm{pos}}^{t}\cup {\mathrm{IoU}}_{\mathrm{refined}}^{t},$$

where ${\mathrm{Cls}}_{\mathrm{pos}}^{t}$ and ${\mathrm{Cls}}_{\mathrm{amb}}^{t}$ are 70-dimensional vectors, denoting the class probability distributions for positive and ambiguous samples. ${\mathrm{Loc}}_{\mathrm{pos}}^{t}$ is a four-dimensional vector representing the bounding box coordinates for positive samples, and ${\mathrm{Loc}}_{\mathrm{refined}}^{t}$ is also a four-dimensional vector, obtained by confidence-weighted averaging of matched positive boxes for ambiguous samples. ${\mathrm{IoU}}_{\mathrm{pos}}^{t}$ is a scalar representing the IoU confidence for positive samples, and ${\mathrm{IoU}}_{\mathrm{refined}}^{t}$ is a scalar computed as the IoU between the refined pseudo-box and the original teacher-predicted box.

For the classification task, we define loss functions for labeled and unlabeled data to guide the student model.

For labeled data, we use the ground truth labels to supervise the classification branch of the student model. The classification loss for supervised data is defined as

(7) $$p_{\sup}={\mathrm{Cls}}_{\sup}^{s}\left[\mathrm{hot}\_\mathrm{index}\left({\mathrm{Cls}}_{\mathrm{gt}}\right)\right],$$

(8) $$L_{\sup}^{\mathrm{cls}}=\mathrm{FL}\;\left(p_{\sup}\right),$$

where $p_{\sup}$ denotes the class probability from the student’s classification output ${\mathrm{Cls}}_{\sup}^{s}$, regarding the class index extracted from the ground-truth one-hot vector ${\mathrm{Cls}}_{\mathrm{gt}}$. hot_index(.) obtains the index of the maximum value in a vector.

For unlabeled data, we adopt pseudo-labels generated by the teacher model, and retain both positive and ambiguous samples after confidence-based filtering. These selected samples are used in consistency learning, as ambiguous samples may still contain meaningful semantic clues. The classification loss for unsupervised data is defined as

(9) $$p_{\mathrm{unsup}}={\mathrm{Cls}}_{\mathrm{unsup}}^{s}\left[\mathrm{hot}\_\mathrm{index}\left({\mathrm{Cls}}_{\mathrm{pseudo}}^{t}\right)\right],$$

(10) $$L_{\mathrm{unsup}}^{\mathrm{cls}}=\mathrm{FL}\;\left(p_{\mathrm{unsup}}\right),$$

where $p_{\mathrm{unsup}}$ denotes the class probability from the student’s classification output ${\mathrm{Cls}}_{\mathrm{unsup}}^{s}$, regarding the class index extracted from the pseudo-label one-hot vector ${\mathrm{Cls}}_{\mathrm{pseudo}}^{t}$, which is provided by the teacher model after filtering.

For the localization task, we separately define the loss for labeled and unlabeled data to ensure stable optimization of the regression branch.

For labeled data, we use the ground-truth bounding boxes as supervision. The regression loss is calculated using the GIoU loss between the student model’s predicted boxes and the ground-truth boxes:

(11) $$L_{\sup}^{\mathrm{loc}}=\mathrm{GIoU}\left({\mathrm{Loc}}_{\mathrm{gt}},{\mathrm{Loc}}_{\sup}^{s}\right),$$

where $B_{\mathrm{gt}}$ is the ground-truth bounding box, and ${\mathrm{Loc}}_{\sup}^{s}$ is the predicted box from the student model for the labeled sample.

For unlabeled data, to enhance the reliability of ambiguous samples, we first examine whether any high-confidence positive samples exist within the local 5×5 neighborhood of each ambiguous box. If no positive samples are found, the ambiguous box itself is re-validated by expanding its grid cell to the same region for local re-prediction. If the re-predicted box achieves an IoU with the initial box higher than 0.9 and its fused confidence exceeds the dynamic threshold $\tau_{\mathrm{pos}}$, it is promoted to a validated box; otherwise, the ambiguous box follows a refinement process.

In the refinement process, ambiguous samples are matched with existing high-confidence positive samples and validated boxes based on three criteria: (1) class consistency—the predicted class of the ambiguous sample must match that of the positive sample; (2) IoU overlap—the IoU between the ambiguous and positive boxes must exceed a predefined threshold; and (3) spatial inclusion—the ambiguous box must be located within the positive box. If all conditions are satisfied, the ambiguous sample is treated as a potential positive and assigned a refined pseudo-box as the regression target.

The refined pseudo-box ${\mathrm{Loc}}_{\mathrm{refined}}^{t}$ is computed by weighted averaging over the matched positive boxes:

(12) $${\mathrm{Loc}}_{\mathrm{refined}}^{t}=\frac{\sum_{j=1}^{N_{\mathrm{match}}}\mathrm{Max}\left({\mathrm{Conf}}_{j}^{t}\right)\cdot {\mathrm{Loc}}_{\mathrm{unsup},j}^{t}}{\sum_{j=1}^{N_{\mathrm{match}}}\mathrm{Max}\left({\mathrm{Conf}}_{j}^{t}\right)},$$

where $\mathrm{Max}({\mathrm{Conf}}_{j}^{t})$ denotes the maximum fused confidence score of the *j*-th matched positive sample, and ${\mathrm{Loc}}_{\mathrm{unsup},j}^{t}$ is the bounding box predicted by the teacher model. $N_{\mathrm{match}}$ represents the number of matched positive samples satisfying the selection criteria.

The regression loss for unlabeled data is then computed as

(13) $$L_{\mathrm{unsup}}^{\mathrm{loc}}=\mathrm{GIoU}\left({\mathrm{Loc}}_{\mathrm{pseudo}}^{t},{\mathrm{Loc}}_{\mathrm{unsup}}^{s}\right),$$

where ${\mathrm{Loc}}_{\mathrm{unsup}}^{s}$ is the student model’s predicted box for the corresponding unlabeled sample.

To guide the auxiliary IoU prediction branch of the teacher model, we introduce an additional binary cross-entropy loss:

(14) $$L_{\mathrm{unsup}}^{\mathrm{iou}}=\mathrm{BCE}\left({\mathrm{Iou}}_{\mathrm{pseudo}}^{t},{\mathrm{Iou}}_{\mathrm{unsup}}^{s}\right),$$

where ${\mathrm{Iou}}_{\mathrm{pseudo}}^{t}$ denotes the IoU confidence scores for the selected pseudo-boxes, and ${\mathrm{Iou}}_{\mathrm{unsup}}^{s}$ is the student model’s predicted IoU for the corresponding samples.

To optimize the student model, we define a total loss that combines both supervised and unsupervised objectives:

(15) $$\begin{array}{cc} L_{\mathrm{total}}\; & =L_{\sup}+L_{\mathrm{unsup}}\\ \; & =\left(L_{\sup}^{\mathrm{cls}}+L_{\sup}^{\mathrm{loc}}\right)+\left(L_{\mathrm{unsup}}^{\mathrm{cls}}+L_{\mathrm{unsup}}^{\mathrm{loc}}+\lambda \cdot L_{\mathrm{unsup}}^{\mathrm{iou}}\right), \end{array}$$

where λ=0.5 controls the relative importance of the IoU prediction loss.

Unlike the student model, the teacher model does not participate in backpropagation. Instead, its parameters are updated via the Exponential Moving Average (EMA) using the student’s weights.

### 3.3. Structured Block-Regularized Neck

To address the common challenge of low detection accuracy for long-distance traffic signs, we propose the Structured Block-Regularized Neck (SBR-Neck) network, which represents an improvement over conventional efficient feature pyramid architectures. The core innovation is twofold, combining multi-scale efficient feature aggregation with a layer-wise block regularization strategy, which introduces spatial-structural perturbations into feature flows. This design effectively suppresses redundancy and improves generalization.

Specifically, we enhance the original pyramid feature fusion pathway by embedding Block Drop Regularize (BDR) units before the input of each layer’s feature channel. This architectural modification systematically prevents the network from becoming overly dependent on local features during training.

**Figure 3:**
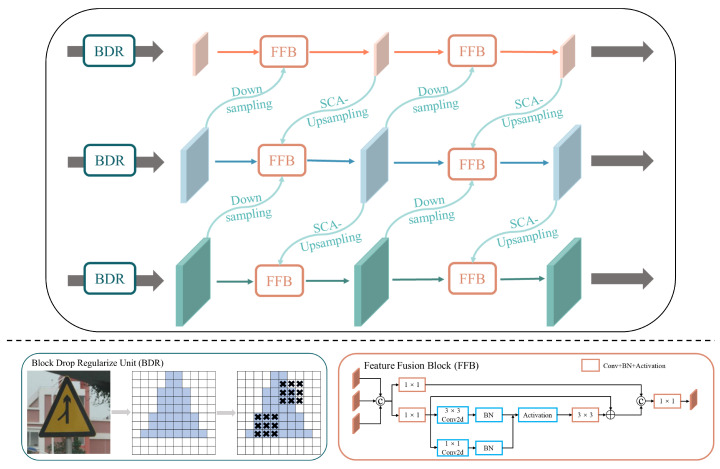
Architecture of the proposed SBR-Neck, which enhances multi-scale feature aggregation by incorporating block-wise spatial perturbations via the Block Drop Regularize (BDR) unit. This strategy improves generalization by suppressing local overfitting and enriches semantic representations for long-distance traffic signs. The feature maps are first downsampled using standard 3×3 convolutions with a stride of 2 to extract hierarchical semantics. For upsampling, we incorporate our proposed SCA-Upsampling, which is detailed in Figure 4.

As shown in Figure 3, the architectural design of SBR-Neck consists of two core components. First, it inherits the multi-level feature-aggregation strategy from classical efficient feature-fusion architectures. Through both horizontal and vertical information flows, the network efficiently integrates features across different semantic hierarchies. Each Feature Fusion Block (FFB) consists of multi-scale convolutional layers and nonlinear transformations. Specifically, each block begins with a 1×1 convolutional layer for channel adjustment, followed by a 3×3 convolution for local spatial feature extraction. Batch normalization and activation functions are then applied to enhance nonlinear representation capabilities. After an additional 3×3 convolution, the sequence ends with a 1×1 convolution to further refine feature representations. This hierarchical design effectively fuses semantic information across scales while balancing computational efficiency and representational capacity by progressive channel compression and expansion.

Second, we introduce the BDR unit preceding the input feature pathway at each layer. For an input feature map $F\in R^{C\times H\times W}$, the BDR unit probabilistically masks contiguous feature blocks with probability *p* and block size $\left(b_{H},b_{W}\right)$. The output features are computed as follows:

(16) $${\tilde{F}}_{i,j}=\left\{\begin{array}{cc} 0, & \mathrm{if}\;(i,j)\in M\\ \frac{F_{i,j}}{1-p}, & \mathrm{otherwise} \end{array}\right.$$

where M is the set of all currently masked spatial block positions, and *p* denotes the block drop probability, namely the probability that a spatial block is randomly set to zero during training. By incorporating BDR prior to each feature fusion stage, the network is compelled to learn independence from specific spatial regions. This mechanism significantly enhances the model’s generalization capability in semi-supervised scenarios by maintaining discriminative power when processing sparse or incomplete information, while effectively suppressing overfitting to local noise patterns.

### 3.4. Spatial-Context-Aware Upsampling

**Figure 4:**
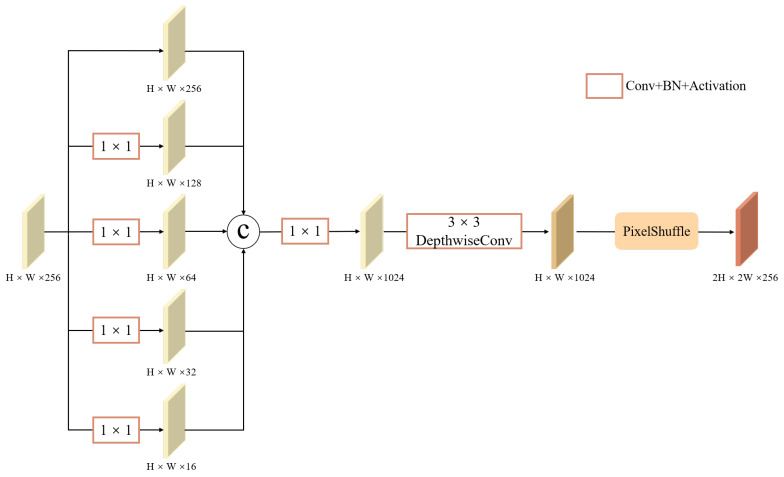
Structure of SCA-Upsampling. The design leverages multi-granularity feature decomposition and depthwise separable convolution to preserve spatial continuity and recover high-frequency details, thereby enhancing localization precision for long-distance traffic signs.

The upsampling process in the object detection task plays a critical role in the accurate localization of multi-scale targets. However, conventional upsampling methods such as bilinear interpolation and transposed convolution often fail to recover high-frequency details, which are essential for precise object boundary representation. To overcome this limitation, we propose a Spatial-Context-Aware Upsampling operation, as illustrated in Figure 4.

First, the input feature map $F_{\mathrm{in}}\in R^{H\times W\times 256}$ is passed through a set of parallel 1×1 convolutional branches, each followed by batch normalization and the SiLU activation function where applicable. These branches generate multi-granularity feature representations with exponentially decreasing channel dimensions:

(17) $$\begin{array}{cc} F_{256} & =F_{\mathrm{in}}\\ F_{128} & =\mathrm{SiLU}({\mathrm{Conv}}_{1\times 1}^{128}\left(\mathrm{BN}\left(F_{\mathrm{in}}\right)\right))\\ \; & \vdots \\ F_{16} & =\mathrm{SiLU}({\mathrm{Conv}}_{1\times 1}^{16}\left(\mathrm{BN}\left(F_{\mathrm{in}}\right)\right)), \end{array}$$

The resulting feature maps are concatenated along the channel dimension to form a unified multi-scale representation. This is followed by a 1×1 convolutional transformation to expand the feature space, facilitating richer feature interactions.

To enhance spatial continuity during upsampling, we introduce a 3×3 depthwise separable convolutional layer before applying the PixelShuffle operation. This layer independently performs spatial filtering on each channel and then fuses channel-wise information, thereby preserving localized spatial structures such as edges and textures.

Finally, the refined feature map undergoes a 1×1 convolution to align the channel dimensions, followed by PixelShuffle-based upsampling. This parameter-free sub-pixel rearrangement effectively increases the spatial resolution, generating an upsampled feature map with improved detail fidelity.

## 4. Experiments

### 4.1. Datasets

The experiments were conducted on the TT100K dataset, which was jointly created and released by Tsinghua University and the Tencent Joint Laboratory. The original dataset includes 128 categories of traffic signs captured across diverse environments. However, a detailed statistical analysis of the dataset reveals a significant class imbalance; specifically, a large number of categories contain very few instances, with some rare classes appearing fewer than 10 times throughout the dataset. These rare categories often lack sufficient samples for effective feature learning, which leads to convergence difficulties during network training and degrades overall detection performance. Consequently, to ensure the reliability of the model, we excluded these rare and extremely low-proportion categories, retaining 84 prevalent traffic sign categories that are critical for urban and expressway driving scenarios.

We constructed a supplementary self-built dataset to address data insufficiency for specific classes and enhance the robustness of the model. The data collection process strictly adhered to the acquisition specifications and environmental diversity standards of the original TT100K benchmark. Specifically, the images were captured using high-definition dashboard cameras and mobile phones, simulating the viewpoints of actual driving scenarios. The collection was conducted primarily during daytime hours, covering a wide range of complex traffic scenes, including urban arterial roads and expressways. This approach ensures that the self-built data maintains consistency with the original dataset in terms of resolution, viewing angles, and background complexity.

To further mitigate overfitting and improve generalization, we employed data augmentation strategies to obtain a final augmented dataset comprising 32,894 images. The dataset is partitioned into training, validation, and test sets with a ratio of 8:1:1.

We also validated the model’s cross-dataset generalization and conducted comparative experiments on the Chinese Traffic Sign Detection Benchmark (CTSDB). Featuring diverse weather and lighting conditions, CTSDB groups traffic signs into three macro-categories: mandatory, prohibitory, and warning. Utilizing this dataset effectively demonstrates the robustness and adaptability of the proposed model to complex background interference in real-world environments.

### 4.2. Implementation Details

In this paper, we present a semi-supervised traffic sign detection method implemented in Python with the PyTorch deep learning framework. The specific software and hardware configurations employed in our experiments are detailed in Table 1.

The proposed method employs YOLOv11s as the detector, with pre-training involving over 300 epochs and semi-supervised training involving 100 epochs. During the training process, the batch size for both unlabeled and labeled data is 16. The Adam optimizer is utilized with a constant learning rate of 0.001 throughout the training procedure. The Exponential Moving Average (EMA) parameter α is set to 0.999, and the loss function weight coefficient is maintained at 1.

### 4.3. Ablation Study

We conducted both quantitative and qualitative ablation studies to validate the proposed modules of our semi-supervised traffic sign detection method.

We conducted a comparative study of three representative YOLO backbones (v5, v8, and v11) under the same semi-supervised setting (Efficient Teacher with 10% labeled data) to validate the choice of YOLOv11. As shown in Table 2, while YOLOv5s achieves the highest inference speed, its detection accuracy is limited to 21.1% due to its relatively simple feature extraction capabilities. Although YOLOv8s improves accuracy to 22.4%, it introduces a significant computational burden, with Parameters increasing by 54% and GFLOPs increasing by 73% compared to v5, leading to a notable drop in FPS. By contrast, YOLOv11s achieves the highest accuracy while maintaining a much lower computational cost than v8s. Thanks to the efficient C3k2 module and attention-driven C2PSA design, YOLOv11s effectively balances model complexity and performance, making it the optimal backbone for our semi-supervised framework where both accuracy and efficiency are paramount.

Table 3 presents the ablation study result for each component of our proposed method with 10% labeled data. We report the mean and standard deviation derived from five folds to demonstrate the stability of the proposed modules. The DC-Fusion module boosts mAP_50_ to 27.9% ± 0.37% and achieves a 4.7% improvement over the baseline. Furthermore, DC-Fusion introduces negligible computational overhead and maintains the inference speed and parameter count at the same level as the baseline.

The integration of SBR-Neck increases mAP_50_ to 27.5% ± 0.26% with a 4.3% gain, and introduces a moderate increase of 1.8 M in Parameters and 5.3 G in FLOPs compared to the baseline. Consequently, this addition causes a decrease in FPS from 62.3 to 55.4, but the significant improvement in detection accuracy justifies this computational cost.

SCA-Upsampling independently yields a mAP_50_ of 25.7% ± 0.38% and adds 0.65 M Parameters to the model. Nevertheless, it incurs a higher computational demand, increasing FLOPs by 6.6 G, due to multi-granularity feature decomposition and depthwise separable convolutions applied to high-resolution feature maps. When all three modules are combined, mAP_50_ reaches 32.1% ± 0.21% and indicates a total improvement of 8.9% over the baseline. The final model operates at 11.85 M Parameters and 33.4 G FLOPs with a speed of 45.8 FPS. These results demonstrate a favorable trade-off between accuracy and efficiency.

**Figure 5:**
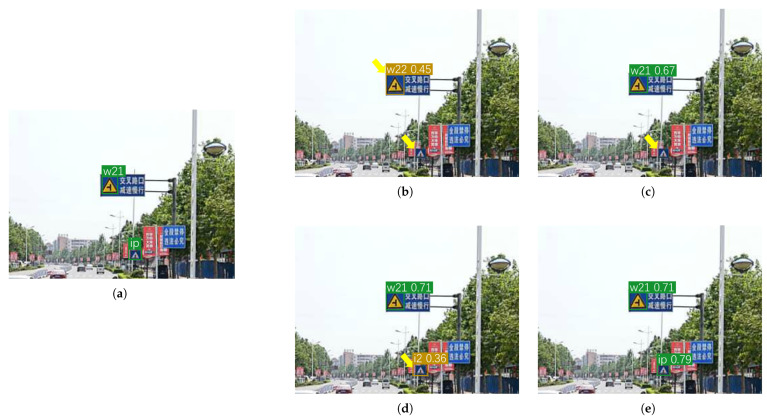
Qualitative analysis of detection results under various ablation configurations. Green boxes indicate correct detections, and brown boxes represent incorrect detections. (**a**) Ground Truth. (**b**) Baseline. (**c**) Baseline + DC-Fusion. (**d**) Baseline + DC-Fusion + SBR-Neck. (**e**) Baseline + DC-Fusion + SBR-Neck + SCA-Upsampling. Yellow arrows indicate missed or false detections.

Figure 5 illustrates the effectiveness of the proposed DC-Fusion, SBR-Neck, and SCA-Upsampling. The baseline model tends to detect false positives—such as misclassified road obstacles and wrong detections—because it relies solely on classification confidence while ignoring localization quality. This limitation also leads to under-confident predictions even when objects are correctly detected, and makes it difficult to capture long-distance targets or avoid errors in large-object detection (see yellow arrows). By contrast, DC-Fusion effectively integrates classification and localization confidences to filter low-quality predictions and enforce spatial consistency checks, thereby reducing positional errors and ensuring geometrically plausible pseudo-boxes. As a result, pseudo-labels are more reliable, and confidence calibration is improved. Furthermore, while DC-Fusion alleviates false positives on large objects, the addition of SBR-Neck further boosts the identification of long-distance targets, and SCA-Upsampling enhances fine-grained spatial representation, ultimately enabling accurate detection across diverse object scales.

To further evaluate the computational overhead introduced by the additional architectural components, we analyze the training cost under the 10% labeled data setting. As shown in Table 4, incorporating DC-Fusion, SBR-Neck, and SCA-Upsampling results in increased resource consumption; specifically, GPU memory consumption increases from 11.4 to 12.8 GB, and the total training time rises from 31.0 to 36.8 h. Although the proposed modules introduce an additional training time and increase the memory footprint, this specific overhead is easily manageable on Tesla T4. More importantly, this small computational investment yields a highly favorable performance efficiency: it achieves a substantial 8.9% improvement in mAP_50_. Therefore, despite the minor increase in training time, the framework remains efficient and practical for robust traffic sign detection.

Table 5 presents the ablation study results of the components in the proposed SCA-Upsampling module. The baseline model achieves only 23.2% mAP_50_, whereas after multi-scale feature concatenation is introduced, the model’s parameter count increases to 638 K, and its mAP_50_ increases to 24.5%. This is because multi-scale feature concatenation separates features across different semantic hierarchies by fusing multi-granularity representations, thereby enhancing cross-scale feature interactions and capturing fine-grained details of traffic signs. Subsequent incorporation of the depthwise separable convolution further increases mAP_50_ to 25.7%, with an increment in parameter number to 647 K. Since the depthwise separable convolution strengthens spatial continuity modeling with its decoupled spatial filtering and channel fusion mechanisms, the model effectively mitigates the degradation in high-frequency detail caused by conventional PixelShuffle methods.

**Figure 6:**
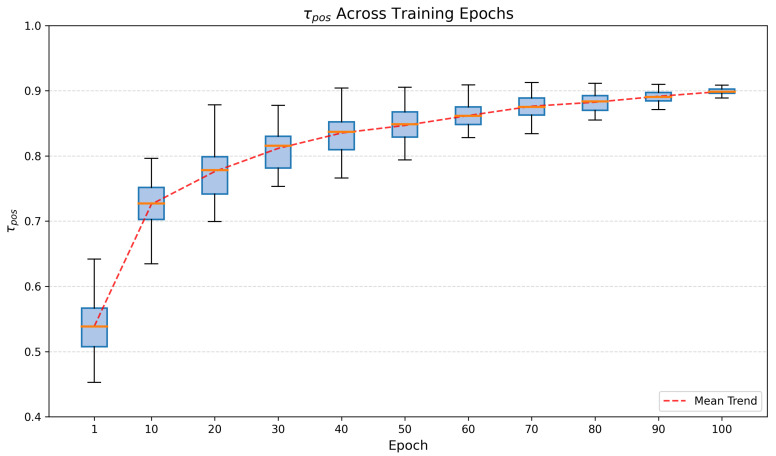
Box-plot visualization of the distribution of the dynamic threshold $\tau_{pos}$ across training epochs. The height of each box represents the inter-batch variance, confirming that $\tau_{pos}$ is dynamically adjusted rather than being a fixed value.

To analyze the stability of the dynamic threshold design, we present a box-plot visualization (Figure 6) to show the distribution of the dynamic threshold $\tau_{pos}$ across different batches at each epoch. $\tau_{pos}$ is usually different across different batches at each training epoch. Unlike traditional methods, where thresholds are fixed or updated in an epoch-wise manner, our proposed strategy computes a unique $\tau_{pos}$ based on the real-time confidence distribution of the current samples. The box plots explicitly illustrate this diversity: at each epoch, the $\tau_{pos}$ values span a range, reflecting the model’s adaptive response to the varying difficulty of different data batches. The relatively large inter-quartile range in the early stages shows that the threshold actively fluctuates to accommodate the model’s unstable predictions. As training converges, the boxes flatten to a single line, demonstrating that while the model becomes stable, the mechanism retains the flexibility to fine-tune the threshold for individual batches, thereby maintaining robustness against hard samples.

To justify the design of SBR-Neck, we analyze the effects of BDR units under different insertion positions and parameter settings. With 10% labeled data, ablation studies are conducted on three configurations: (1) insertion between the backbone and neck, (2) insertion between the neck and prediction heads, and (3) insertion at both junctions. The results are summarized in Table 6 and show that applying BDR only between the backbone and neck yields the best performance.

This placement leverages the backbone’s multi-scale, high-resolution features before fusion, where structured dropout suppresses redundancy while preserving spatial correlations crucial for localization. Perturbation after the neck may corrupt refined semantic features, and dual-position insertion overly disrupts feature continuity.

Under the 10% labeled data setting, in this study, we conduct ablation experiments on two critical parameters in BDR, the drop probability *p* and block size $\left(b_{H},b_{W}\right)$, as presented in Table 7. Experimental results demonstrate that the model achieves optimal performance when the block size is configured as 5×5 and the drop probability is set to 0.2. The 5×5 masking region effectively compels the model to focus on inter-region feature correlations by structured dropping, while avoiding semantic discontinuity caused by excessive masking. Concurrently, the 0.2 drop probability establishes a dynamic balance between regularization intensity and feature preservation, ensuring sufficient regularization strength without compromising critical feature representations.

**Figure 7:**
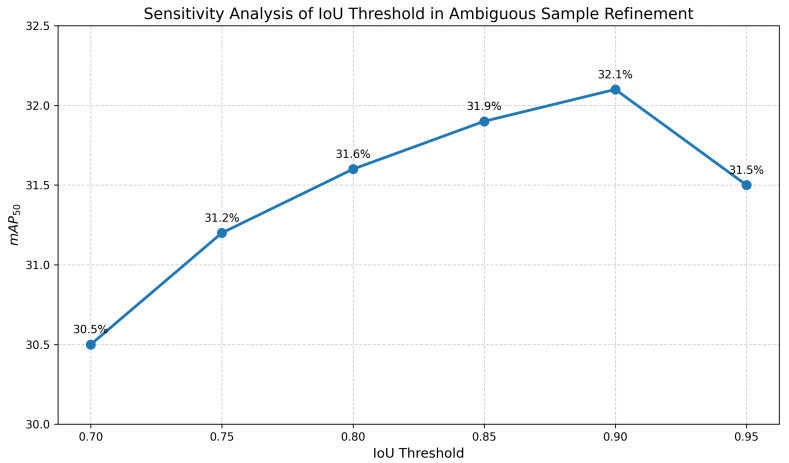
Sensitivity analysis of the IoU threshold in ambiguous sample refinement. The curve shows that the detection of mAP_50_ peaks occurs when the threshold is set to 0.9, validating our parameter choice.

To investigate the impact of IoU threshold choices on the ambiguous sample refinement process (Equation (12)), we conducted a sensitivity analysis under the 10% labeled-data setting. The IoU threshold determines the strictness of matching ambiguous samples with high-confidence positive samples. As shown in Figure 7, we evaluated the model performance by varying the threshold from 0.70 to 0.95.

The experimental results demonstrate an inverted U-shaped trend. When the threshold is set to a lower value, the model achieves a lower mAP_50_ of 30.5% and 31.2%, respectively. This performance degradation is attributed to the inclusion of low-quality matches, which introduces noise into the pseudo-labels and misguides the student model. Conversely, an overly strict threshold leads to a slight performance drop to 31.5%, as it filters out potentially useful ambiguous samples that could contribute to training. The optimal performance of 32.1% is achieved when the threshold is set to 0.90, which strikes a balance between filtering noisy samples and mining informative pseudo-labels. Based on this observation, we set the IoU threshold to 0.90 for all subsequent experiments.

Overall, these experimental results comprehensively demonstrate the effectiveness of each module in our proposed method.

### 4.4. Comparison with Other Upsampling Methods

Table 8 demonstrates the performance comparison between the proposed SCA-Upsampling and other upsampling methods under the 10% labeled data setting. The experimental results reveal that traditional upsampling methods achieve mAP_50_ scores below 24%, with negligible parameter counts or minimal adjustments via simple convolutional layers. This indicates that relying solely on interpolation or shallow convolutions fails to effectively restore high-frequency detail features, resulting in blurred edges for long-distance targets and insufficient localization accuracy. By contrast, the Meta-Upscale method [41], which optimizes the upsampling process by meta-learning, improves mAP_50_ to 24.8%. However, its parameter count and moderate FPS exhibit limited cost–performance efficiency relative to the performance gain, and it does not explicitly model multi-scale contextual information. The proposed SCA-Upsampling addresses these limitations by incorporating multi-granularity feature decomposition and enhancing spatial continuity with depthwise separable convolutions. Notably, SCA-Upsampling achieves the highest detection accuracy while maintaining real-time inference speed, achieving a favorable balance between performance and efficiency.

### 4.5. Comparison with the State-of-the-Art Methods

To comprehensively validate the effectiveness of our proposed method, we conducted a comparative evaluation against recent state-of-the-art semi-supervised object detection methods. The comparison includes end-to-end frameworks such as Omni-DETR [43] and Semi-DETR [39], two-stage approaches like STAC [37] and PseCo [44], and one-stage detectors including Efficient Teacher [45] and Dense Teacher [46]. Table 9 presents the performance using mAP_50_ across varying labeled data ratios, while Table 10 details the mAP_50:95_ metric under the same experimental settings to provide a deeper analysis of localization accuracy. All reported results represent the mean and standard deviation over five folds to ensure a rigorous comparison.

The quantitative results demonstrate that our proposed method consistently outperforms all compared approaches across the 1%, 2%, 5%, and 10% labeled data settings. As shown in Table 9, our framework achieves higher mAP_50_ scores than the leading end-to-end method Semi-DETR, with improvements of 0.68%, 1.6%, 2.2%, and 2.7% across the four settings, respectively. Furthermore, our model shows significant gains over PseCo, which represents the top performance among two-stage methods. When compared to the one-stage baseline Efficient Teacher and the closest competitor Dense Teacher, our method exhibits consistent superiority and achieves a mAP_50_ of 32.1% under the 10% setting.

Table 10 further highlights the robustness of our approach in terms of high-precision localization. Although transformer-based methods like Semi-DETR typically excel in boundary regression due to their global attention mechanisms, our method achieves a mAP_50:95_ of 19.8% and surpasses Semi-DETR by 1.7% under the 10% labeled data setting. This result indicates that the integration of the Dual Confidence Fusion Module and Spatial-Context-Aware Upsampling effectively enhances the geometric quality of the predicted bounding boxes. Moreover, our method maintains a real-time inference speed of 45.8 FPS, which is significantly faster than the 9.1 FPS of Semi-DETR and comparable to other one-stage detectors. These findings confirm that our approach achieves an optimal balance between detection accuracy and computational efficiency.

Although our method’s FPS is slightly lower than some one-stage detectors, it significantly exceeds all end-to-end methods in inference speed and remains faster than most two-stage counterparts. This demonstrates a strong trade-off between detection accuracy and computational efficiency, which is crucial for real-world deployment.

**Figure 8:**
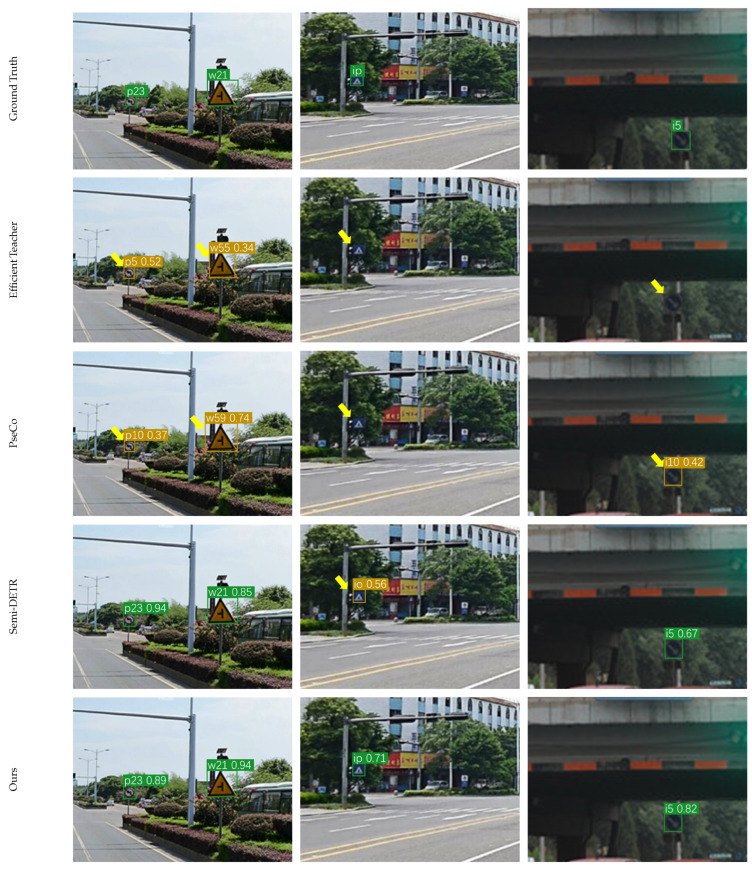
Visual comparison of detection results among state-of-the-art semi-supervised object detection methods and the proposed approach. Our method demonstrates superior robustness under challenging conditions and effectively detects long-distance signs, indicating the advantages of DC-Fusion, SBR-Neck, and SCA-Upsampling. Green boxes indicate correct detections, brown boxes represent incorrect detections, and yellow arrows indicate missed or false detections.

Figure 8 shows visual comparisons among representative SSOD methods, where yellow arrows indicate missed or incorrect detections. In this figure, column (a) shows cases where different scales of traffic signs coexist, column (b) illustrates cases involving long-distance targets, and column (c) shows detection results under low-light conditions. As illustrated, methods such as Efficient Teacher and Unbiased Teacher often fail to detect long-distance traffic signs, especially under low-light conditions. In these challenging cases, traffic signs are completely missed.

By contrast, our method shows improved robustness. It reliably detects traffic signs under low-light conditions, while substantially reducing false positives. For long-distance signs, the predicted boxes are more accurate and stable. These improvements are primarily due to the proposed DC-Fusion strategy, which filters unreliable pseudo-labels, and the Structured Block-Regularized Neck, which guides the model to focus on discriminative spatial features.

To address the concern regarding dataset diversity, we further evaluate the proposed method on the CTSDB dataset under the 10% labeled setting. The same training protocol and hyperparameter configuration are adopted to ensure fair comparison.

As shown in Table 11, our method achieves 35.3% mAP_50_, outperforming all competing methods. Compared with the strongest baseline, Semi-DETR, our approach yields a performance gain of 3.7%.

## 5. Conclusions

Current semi-supervised traffic sign detection methods often suffer from low-quality pseudo-labels due to the limited performance of teacher models trained on small labeled datasets, which results in false positives. To address these challenges, we propose a semi-supervised traffic sign detection framework that integrates the Dual Confidence Fusion Module (DC-Fusion) and the Structured Block-Regularized Neck. DC-Fusion improves pseudo-label quality by jointly evaluating classification and localization confidences. The proposed Structured Block-Regularized Neck network (SBR-Neck) reduces local overfitting with structured feature perturbation and improves feature generalization across scales. Additionally, Spatial-Context-Aware Upsampling (SCA-Upsampling) enhances spatial detail recovery and localization accuracy with multi-granularity feature decomposition and depthwise separable convolutions. These improvements significantly reduce missed detections, especially for long-distance traffic signs, and demonstrate strong performance under limited supervision.

Limitations and Future Work: While our method achieves substantial performance gains, several limitations remain:

1. Training Efficiency: The integration of DC-Fusion, SBR-Neck, and SCA-Upsampling increases model complexity and computational overhead. Future work will explore lightweight architectural designs and dynamic computation strategies to enhance training efficiency.
2. Domain Adaptation: Distributional discrepancies between labeled and unlabeled data in the early training stages can lead to model instability. Incorporating domain adaptation techniques could mitigate this challenge and enhance model robustness.

Future research will focus on addressing these limitations and further exploring the generalization capabilities of the proposed framework in real-world applications such as autonomous driving and industrial inspection. Our ultimate objective is to develop a more efficient and robust semi-supervised object detection system adaptable to diverse application scenarios.

## Figures and Tables

**Table 1:** Hardware and software configurations in the experimental setup.

Component	Specification
Operating System	Ubuntu 16.04
CPU	Intel(R) Xeon(R) Gold 5218 CPU @2.30 GHz
Memory	64 GB
GPU	Tesla T4 ×2
VRAM	15 GB ×2
Python	3.7
PyTorch	1.13.1

**Table 2:** Comparison of different backbones within the semi-supervised framework (10% labeled data). All models use the Efficient Teacher baseline without additional modules.

Backbone	Params (M)	GFLOPs	mAP_50_	FPS
YOLOv5s	7.2	16.5	21.1%	66.7
YOLOv8s	11.1	28.6	22.4%	55.8
YOLOv11s	9.4	21.5	23.2%	62.3

**Table 3:** Ablation study on the proposed DC-Fusion, SBR-Neck, and SCA-Upsampling based on the Efficient-Teacher baseline. The results are the average of all five folds ± standard deviation. ✓ indicates that the corresponding module is enabled in the ablation experiment.

DC-Fusion	SBR-Neck	SCA-Upsampling	Params	GFLOPs	mAP_50_	FPS
			9.40 M	21.5	23.2% ± 0.45%	62.3
✓			9.40 M	21.5	27.9% ± 0.37%	62.3
	✓		11.20 M	26.8	27.5% ± 0.26%	55.4
		✓	10.05 M	28.1	25.7% ± 0.38%	52.5
✓	✓		11.20 M	26.8	28.8% ± 0.25%	55.6
✓		✓	10.05 M	28.1	27.9% ± 0.37%	52.3
	✓	✓	11.85 M	33.4	28.1% ± 0.26%	45.7
✓	✓	✓	11.85 M	33.4	32.1% ± 0.21%	45.8

**Table 4:** Comparison of training cost between the baseline and proposed method under the 10% labeled data setting.

Method	Memory	Time/Epoch	Total Time
Efficient Teacher (Baseline)	11.4 GB	18.6 min	31.0 h
+DC-Fusion	11.8 GB	19.4 min	32.3 h
+SBR-Neck	12.5 GB	21.2 min	35.3 h
Our Proposed (All Modules)	12.8 GB	22.1 min	36.8 h

**Table 5:** Detailed ablation study of SCA-Upsampling based on the Efficient-Teacher baseline, analyzing the contribution of multi-scale feature concatenation and depthwise separable convolutions.

Components	Params	mAP_50_
Baseline	0	23.2%
+Multi-scale Feature Concatenation	638 K	24.5%
+Depthwise Separable Convolution	647 K	25.7%

**Table 6:** Comparative study of different insertion points for BDR unit within the backbone–neck–head pipeline. ✓ indicates that the corresponding module is enabled in the ablation experiment.

Backbone and Neck	Neck and Head	mAP_50_	Recall	FPR
✓		27.5%	79.3%	9.8%
	✓	26.8%	78.2%	10.2%
✓	✓	26.1%	77.4%	11.1%

**Table 7:** Parameter sensitivity analysis of the BDR unit.

$\left(b_{H},b_{W}\right)$	*p*	mAP_50_
	0.1	26.9%
3×3	0.2	26.8%
	0.3	26.5%
	0.1	27.1%
5×5	0.2	27.5%
	0.3	27.3%
	0.1	26.9%
7×7	0.2	27.2%
	0.3	27.0%

**Table 8:** Comparative evaluation of the proposed SCA-Upsampling against conventional upsampling methods.

Method	Params	mAP_50_	FPS
Bilinear	0	23.2%	62.3
Bilinear + Conv	590 k	23.4%	55.1
Nearest + Conv	590 k	23.4%	55.2
Deconv [42]	590 k	23.1%	55.7
Meta-Upscale [41]	656 k	24.8%	50.8
SCA-Upsampling (Ours)	647 k	25.7%	52.5

**Table 9:** Comparison of mAP_50_ between the proposed method and recent state-of-the-art SSOD methods. All results are the average of all five folds ± standard deviation.

Method	1%	2%	5%	10%	FPS
Omni-DETR [43]	9.07% ± 0.42%	15.1% ± 0.38%	20.1% ± 0.31%	28.7% ± 0.27%	10.7
Semi-DETR [39]	9.72% ± 0.47%	16.2% ± 0.41%	21.5% ± 0.34%	29.4% ± 0.29%	9.1
STAC [37]	5.54% ± 0.36%	7.36% ± 0.32%	12.9% ± 0.28%	21.2% ± 0.24%	14.4
Unbiased Teacher [27]	6.81% ± 0.39%	12.5% ± 0.34%	16.7% ± 0.29%	24.5% ± 0.26%	16.8
PseCo [44]	8.04% ± 0.35%	14.8% ± 0.30%	19.1% ± 0.27%	27.8% ± 0.23%	15.7
Humble Teacher [38]	6.48% ± 0.33%	9.31% ± 0.28%	15.2% ± 0.26%	24.5% ± 0.22%	17.8
Efficient Teacher [45]	7.33% ± 0.28%	12.5% ± 0.24%	15.7% ± 0.21%	21.1% ± 0.19%	66.7
Unbiased Teacher v2 [47]	7.19% ± 0.30%	9.82% ± 0.26%	16.2% ± 0.23%	23.5% ± 0.20%	48.8
One Teacher [48]	7.47% ± 0.29%	11.4% ± 0.25%	15.8% ± 0.22%	24.7% ± 0.21%	46.7
Dense Teacher [46]	8.03% ± 0.27%	12.8% ± 0.23%	16.9% ± 0.21%	25.1% ± 0.19%	48.6
Ours	10.4% ± 0.35%	17.8% ± 0.27%	23.7% ± 0.22%	32.1% ± 0.21%	45.8

**Table 10:** Comparison of mAP_50:95_ between the proposed method and recent state-of-the-art SSOD methods. All results are the average of all five folds ± standard deviation.

Method	1%	2%	5%	10%
Omni-DETR [43]	5.40% ± 0.25%	9.33% ± 0.23%	12.25% ± 0.19%	17.33% ± 0.16%
Semi-DETR [39]	5.70% ± 0.28%	9.50% ± 0.24%	12.52% ± 0.20%	18.07% ± 0.18%
STAC [37]	3.35% ± 0.22%	4.48% ± 0.19%	7.49% ± 0.16%	13.12% ± 0.15%
Unbiased Teacher [27]	4.18% ± 0.24%	7.36% ± 0.20%	9.81% ± 0.17%	14.39% ± 0.15%
PseCo [44]	4.76% ± 0.21%	8.89% ± 0.18%	11.41% ± 0.16%	16.45% ± 0.14%
Humble Teacher [38]	3.92% ± 0.20%	5.45% ± 0.16%	8.99% ± 0.15%	14.57% ± 0.13%
Efficient Teacher [45]	4.39% ± 0.17%	7.64% ± 0.15%	9.23% ± 0.12%	12.67% ± 0.11%
Unbiased Teacher v2 [47]	4.34% ± 0.18%	5.71% ± 0.15%	9.79% ± 0.14%	13.79% ± 0.12%
One Teacher [48]	4.35% ± 0.17%	7.04% ± 0.15%	9.77% ± 0.14%	15.12% ± 0.13%
Dense Teacher [46]	4.76% ± 0.16%	7.47% ± 0.13%	10.26% ± 0.13%	15.00% ± 0.11%
Ours	6.08% ± 0.20%	10.68% ± 0.16%	13.78% ± 0.13%	19.79% ± 0.13%

**Table 11:** Performance comparison on the CTSDB dataset using 10% labeled data. All results are the average of all five folds ± standard deviation.

Method	mAP_50_	mAP_50:95_
Omni-DETR [43]	30.8% ± 0.31%	17.9% ± 0.24%
Semi-DETR [39]	31.6% ± 0.28%	18.6% ± 0.22%
STAC [37]	23.5% ± 0.26%	13.4% ± 0.20%
Unbiased Teacher [27]	27.4% ± 0.24%	15.8% ± 0.18%
PseCo [44]	29.2% ± 0.22%	16.9% ± 0.17%
Humble Teacher [38]	27.8% ± 0.21%	16.1% ± 0.16%
Efficient Teacher [45]	24.6% ± 0.20%	14.1% ± 0.15%
Unbiased Teacher v2 [47]	26.9% ± 0.19%	15.5% ± 0.14%
One Teacher [48]	28.1% ± 0.18%	16.3% ± 0.13%
Dense Teacher [46]	29.4% ± 0.17%	17.1% ± 0.12%
Ours	35.3% ± 0.23%	21.4% ± 0.16%

## Data Availability

The data presented in this study are available on request from the corresponding author. The data are not publicly available due to privacy restrictions.
